# Robust Topographical Micro-Patterning of Nanofibrillar Collagen Gel by In Situ Photochemical Crosslinking-Assisted Collagen Embossing

**DOI:** 10.3390/nano10122574

**Published:** 2020-12-21

**Authors:** Hyeonjun Hong, Dong Sung Kim

**Affiliations:** Department of Mechanical Engineering, Pohang University of Science and Technology (POSTECH), 77 Cheongam-ro, Nam-gu, Pohang, Gyeongbuk 37673, Korea; membrain@postech.ac.kr

**Keywords:** cell microenvironments, nanofibrillar collagen gel, micro-patterning, collagen compression, collagen embossing, photochemical crosslinking, cell culture scaffold, anisotropic topography

## Abstract

The topographical micro-patterning of nanofibrillar collagen gels is promising for the fabrication of biofunctional constructs mimicking topographical cell microenvironments of in vivo extracellular matrices. Nevertheless, obtaining structurally robust collagen micro-patterns through this technique is still a challenging issue. Here, we report a novel in situ photochemical crosslinking-assisted collagen embossing (IPC-CE) process as an integrative fabrication technique based on collagen compression-based embossing and UV–riboflavin crosslinking. The IPC-CE process using a micro-patterned polydimethylsiloxane (PDMS) master mold enables the compaction of collagen nanofibrils into micro-cavities of the mold and the simultaneous occurrence of riboflavin-mediated photochemical reactions among the nanofibrils, resulting in a robust micro-patterned collagen construct. The micro-patterned collagen construct fabricated through the IPC-CE showed a remarkable mechanical resistivity against rehydration and manual handling, which could not be achieved through the conventional collagen compression-based embossing alone. Micro-patterns of various sizes (minimum feature size <10 μm) and shapes could be obtained by controlling the compressive pressure (115 kPa) and the UV dose (3.00 J/cm^2^) applied during the process. NIH 3T3 cell culture on the micro-patterned collagen construct finally demonstrated its practical applicability in biological applications, showing a notable effect of anisotropic topography on cells in comparison with the conventional construct.

## 1. Introduction

Cell microenvironments are complex and dynamic networks in biological tissues. Such networks consist of an extracellular matrix (ECM), soluble macromolecules, interstitial fluid, and neighboring cells, which greatly affect the cell behavior (e.g., in terms of adhesion, migration, proliferation, and differentiation) [1]. Above all, the ECM of various tissues is characterized by distinct topographical micro-patterns organized by collagen nanofibrils. Examples of these structures are the micro-pit structure of bones (≈30–50 μm in diameter) [2], the honeycomb-like micro-network of muscles (i.e., the endomysium; ≈10–60 μm in diameter) [3], and the micro-grooved structure of corneas (i.e., the limbal epithelial stem cell niche; ≈15–150 μm in width) [4]. The tissue-specific topographical features of the ECM play a crucial role in physiological (e.g., tissue development and homeostasis) and pathological (e.g., cancer metastasis and wound healing) processes through contact guidance or geometrical confinement, which are based on cell–matrix interactions [5,6,7,8]. Substantial efforts have been devoted in the field of tissue engineering to achieve topographical micro-patterns mimicking those of native tissues and manipulate cell functions in the development of in vitro tissue models or scaffolds for in vivo applications [9]. Natural polymers (e.g., collagen, fibrin, alginate, and chitosan), due to their excellent bio-functionalities, have been widely utilized for constructing topographical micro-patterns [10,11,12,13]. 

Collagen gel, which is obtained by the self-assembly of a major biochemical component of native ECM, provides the opportunity of realizing topographical micro-patterns more similar to physiological ones, given that it can provide both in vivo ECM-like nanofibrillar architectures and specific amino acid sequences on them, which are known as important determinants for physiological cell functions. [14,15]. These advantageous features of collagen gel have stimulated the development of various methods (e.g., chemical crosslinking [16,17,18], surface coating [19], air-drying [10], and collagen compression-based embossing [20,21]) to fabricate micro-patterned collagen constructs mimicking the complex features of native ECM. Among all of these methods, collagen compression-based embossing showed potential in the production of micro-patterned collagen constructs, due to its facile and rapid fabrication procedure compared to conventional approaches: it requires only one step of mechanical compression to achieve the compaction of collagen nanofibrils into a micro-patterned master mold with the dehydration [18]. Moreover, the micro-patterns over the surface of the fabricated construct typically present a dense collagen nanofibrillar architecture similar to that of native tissues (e.g., skin dermis, muscle, and cornea) [3,20,22,23]. Nonetheless, the practical and wide-utilization of this approach in biological applications is limited, because fabricated micro-patterns can be readily disrupted by rehydration in aqueous solutions (e.g., cell culture medium or phosphate-buffered saline; PBS) and manual handling. Such a disruption of the micro-patterns, in turn, may lead to a loss of topographical effect on cells. 

Here, we developed a novel in situ photochemical crosslinking-assisted collagen embossing (IPC-CE) process, based on a conventional collagen compression-based embossing and UV-riboflavin crosslinking to fabricate a nanofibrillar collagen construct with robust micro-patterns. The IPC-CE allowed not only compacting the collagen nanofibrils into a polydimethylsiloxane (PDMS) master mold but also applying UV light to the collagen gel for inducing photochemical crosslinking among the collagen nanofibrils in a simultaneous manner, thereby producing a mechanically robust micro-patterned collagen construct. The micro-patterns of the fabricated construct were highly robust enough to maintain its structure from the rehydration in the medium for the cell culture and manual handling. Various sizes and types of the micro-patterns with favorable transcription quality could be generated through modulating processing parameters (i.e., compressive pressure and UV dose) in the IPC-CE process with the PDMS master molds. The structural robustness of the micro-patterns was finally demonstrated by the in vitro cell culture, which exhibited that the alignment and elongation of the cells were promoted by the micro-patterns on the construct, while the collagen constructs fabricated by the conventional compression-based embossing could not achieve the topographical effect on the cells due to the rehydration.

## 2. Materials and Methods

### 2.1. Preparation of the Micro-Patterned PDMS Master Mold

A micro-patterned PDMS master mold was prepared by UV photolithography and through the subsequent PDMS replica molding, as described in previous studies [24,25]. Briefly, micro-ridge patterns were created on a silicon wafer with a negative photoresist (SU-8 2050, Kayaku Advanced Materials, Inc., Westborough, MA, USA) following manufacturer’s instructions. This silicon wafer was successively utilized as a template for the micro-patterned PDMS master mold. A mixture of PDMS base and curing agent (weight ratio = 10:1; Dow Corning Corp., Midland, MI, USA) was poured on the silicon template and cured at 60 °C for >4 h. Various types of the PDMS master molds with negative micro-ridge patterns were prepared in this study. Their micro-cavities were 100/100/40 μm, 80/80/40 μm, 60/60/40 μm, or 20/20/40 μm in width/spacing/depth. 

### 2.2. Description of the IPC-CE Process

**Figure 1:**
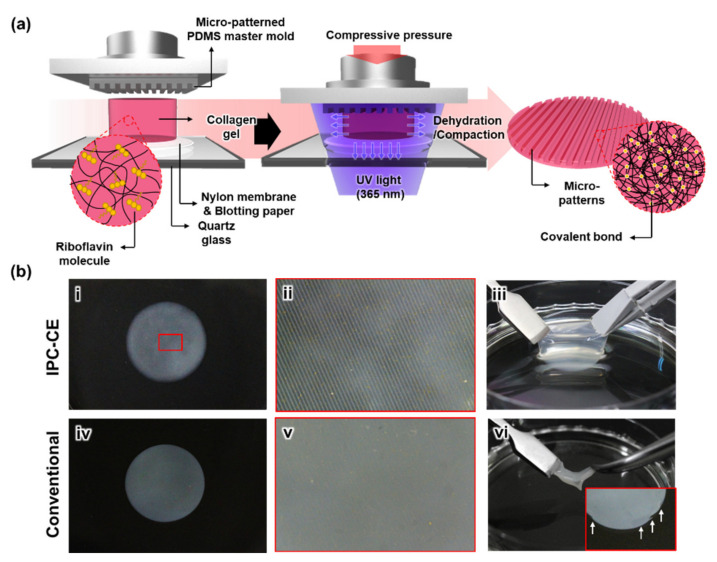
Fabrication of the in situ photochemical crosslinking-assisted collagen embossing (IPC-CE) construct. (**a**) Schematic illustration of the IPC-CE process. (**b**) Photographs of (**i**) the IPC-CE construct and (**ii**) its magnified surface. (**iii**) Manual handling of the IPC-CE construct. (**iv**) The conventional construct and (**v**) its magnified surface. (**vi**) Structural weakness of conventional construct for manual handling. White arrows in inset indicated damages induced by manual handling.

A neutralized collagen solution (final concentration, 3 mg/mL) was prepared by adding 1 M NaOH to a rat tail type I collagen solution (telocollagen, >3.5 mg/mL in 0.02 N acetic acid; Corning Inc., Corning, NY, USA) and diluting all of it with 1× Dulbecco’s modified Eagle’s medium (DMEM; Hyclone Laboratories Inc., Logan, UT, USA). Then, a riboflavin (Sigma-Aldrich, St. Louis, MO, USA) powder was solubilized in the neutralized collagen solution by gentle mixing at a concentration of 0.01% (*w*/*v*). Afterward, the solution was incubated in a humidified cell culture incubator at 37 °C for 30 min to form a gel with the aid of a polymethyl methacrylate (PMMA) template with a hole (15 mm in diameter and 5 mm in height). For the IPC-CE process, the collagen gel was positioned between the micro-patterned PDMS master mold and a set of nylon membranes (CHMLAB Group, Barcelona, Spain) and blotting paper (Hyundai Micro, Seoul, Korea), and they were placed on the bottom quartz glass plate of a customized collagen compression system (Figure 1a and Figure S1). Subsequently, a compressive pressure was applied to the collagen gel through the linear movement of a top plate driven by a motorized linear stage at a compression rate of <50 μm/s and a maximum compressive pressure between 2 and 115 kPa. In the meantime, the collagen gel was exposed to UV light from the bottom side using a UV irradiation system (wavelength = 365 nm; Panasonic Corp., Osaka, Japan) at an intensity of 2.5 mW/cm^2^. The whole process resulted in the production of a micro-patterned collagen construct. The total cycle time of the IPC-CE process varied between 6 and 20 min, depending on the UV dose applied to the collagen gel (between 0.90 and 3.00 J/cm^2^). For comparison purposes, a conventional collagen compression-based embossing without photochemical crosslinking was also performed under compressive pressures of 2 and 115 kPa, generating two different micro-patterned collagen constructs (Figure S1). From this point onward, the micro-patterned collagen constructs fabricated through the IPC-CE process and the conventional collagen compression-based embossing will be simply called the “IPC-CE construct” and “conventional construct”, respectively.

### 2.3. Parametric Effects of the IPC-CE Process

The effects of the processing parameters (i.e., compressive pressure and UV dose) of the IPC-CE process on the formation and maintenance of the micro-ridge patterns (100/100/40 μm in width/spacing/height) were evaluated from top-view microscopic images of the fabricated construct. The collagen construct samples were prepared under different processing conditions (as described above) and stored in a PBS solution for at least 10 min to allow rehydration. Then, each sample was fixed with a 4% paraformaldehyde solution (which was left to act for 15 min), washed more than three times, and positioned on a glass slide with the micro-patterned surface facing upward; finally, the liquid covering the surface was removed with blotting paper. A top image of each sample loaded on the glass slide was taken by using an optical microscope (Eclipse 80i; Nikon Instruments Inc., Tokyo, Japan) and further observed after air-drying overnight. The micro-ridge patterns were quantitatively assessed by measuring their widths in the images through ImageJ (NIH, Bethesda, MD, USA). 

### 2.4. Nanofibrillar Architecture of the IPC-CE Construct

The nanofibrillar architectures of the samples were investigated by using a scanning electron microscope (SEM; SU6600, Hitachi, Tokyo, Japan). The samples stored in the PBS were first fixed with a 4% paraformaldehyde solution for 15 min and then washed three times with deionized (DI) water. Then, the fixed sample was dehydrated by adding a graded ethanol series (30, 50, 70, 90, and 100% ethanol in DI water for 15 min each) and dried using 50% hexamethyldisilazane (HMDS; Sigma-Aldrich, St. Louis, MO, USA) in ethanol for 15 min and 100% HMDS overnight. Finally, the dried samples were examined by SEM after sputter-coating with Pt at 15 mA for 120 s. 

### 2.5. Structural Robustness and Topographical Effect of Micro-Patterns in an in Vitro Cell Culture

The structural robustness of the replicated collagen micro-patterns, which were prepared from the PDMS master mold with micro-ridge patterns (100/100/40 μm in width/spacing/height), was investigated by observing time-dependent topographical changes in the micro-patterns caused by rehydration in the PBS solution. The micro-patterns were observed with the same method as described above: (1) right after the fabrication of the collagen construct; (2) after the rehydration in the PBS solution for >10 min. To practically evaluate the topographical effect on cells of our robust collagen micro-patterns, we conducted an in vitro culture of NIH 3T3 mouse embryonic fibroblast cells (Korean Cell Line Bank, Seoul, Korea). The NIH 3T3 cells were maintained in high-glucose DMEM supplemented with 10% fetal bovine serum (FBS; Hyclone Laboratories Inc., Logan, UT, USA) and 1% penicillin/streptomycin (Hyclone Laboratories Inc., Logan, UT, USA). The cells were seeded on each sample at a density of 1 × 10^4^ cells/cm^2^, and the medium was changed every day. After 2 days of cell culture, the cells were fixed with 4% paraformaldehyde for 15 min, permeabilized with 0.3% Triton X-100 in PBS at 4 °C for 15 min, and treated with 1% bovine serum albumin at room temperature for 1 h for immunofluorescence staining. Then, the cells were stained by using tetramethylrhodamine isothiocyanate (TRITC)-conjugated phalloidin (Sigma-Aldrich, St. Louis, MO, USA) and 4′,6-diamidino-2-phenylindole (DAPI; Invitrogen, Carlsbad, CA, USA) at room temperature for 60 and 5 min, respectively. The cell alignment was quantitatively analyzed based on images of the DAPI-stained cells by measuring both the aspect ratio and the orientation angle of the major axis of the nuclei from the centerline of the micro-ridge pattern. 

## 3. Results

### 3.1. Fabrication of the IPC-CE Construct

The newly developed IPC-CE process could successfully produce an IPC-CE construct with micro-ridge patterns by the simultaneous applications of collagen compression (using a PDMS master mold) and in situ photochemical crosslinking (for less than 20 min). Micro-patterns were well replicated on the surface of the IPC-CE construct and were observable even by the naked eye (Figure 1(bi,bii)). Notably, the IPC-CE construct stably maintained its macroscopic structure and surface micro-patterns (without deformation nor damage) when subjected to immersion in the PBS solution or cell culture medium and manual handling (Figure 1(biii)). Meanwhile, the micro-patterns of the conventional construct, which were subjected to a compressive pressure of 2 kPa, were difficult to distinguish (Figure 1(biv,bv)); notably, their macroscopic structure was very weak and prone to damage when manually handled with a tweezer (Figure 1(bvi)). 

### 3.2. Effects of the Processing Parameters in the IPC-CE Process

**Figure 2:**
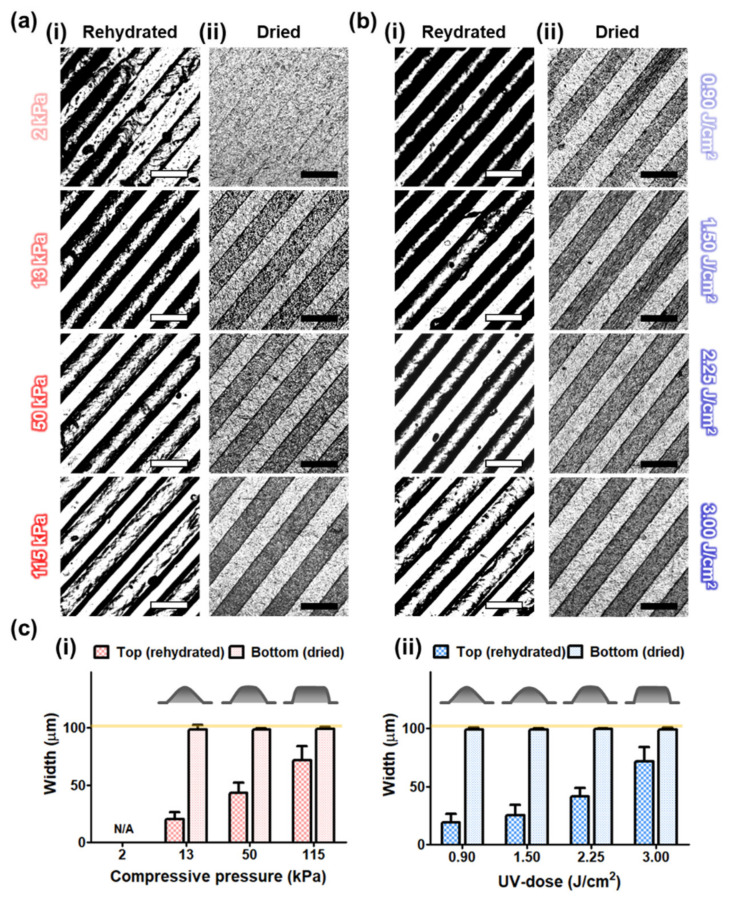
Effects of processing parameters on micro-ridge patterns. Microscopic images of the IPC-CE constructs for (**a**) different compressive pressures at the same UV dose of 3.00 J/cm^2^ and (**b**) different UV doses at same compressive pressure of 115 kPa in (**i**) rehydrated and (**ii**) dried state. All scale bars are 200 μm. (**c**) Quantitative measurement of micro-ridge pattern width for rehydrated and dried IPC-CE construct according to (**i**) compressive pressure and (**ii**) UV-dose.

The microscopic observations of the IPC-CE construct indicated that the applied compressive pressure and the UV dose both affected the formation of micro-ridge patterns. In the rehydrated state, the IPC-CE construct showed that a partial and poor replication of the micro-ridge patterns when obtained at the lowest compressive pressure tested (2 kPa) and a UV dose of 3.0 J/cm^2^ (Figure 2(ai)). Instead, a higher compressive pressure (13 kPa) allowed the replication of the micro-ridge patterns over the entire surface of the construct at the same UV dose (without discontinuities). The micro-ridge patterns were generally found to have a narrow top (shown in bright white) and rounded edges (shown in dark black; please note that shadows were generated by the remaining liquid), which may be attributed to an incomplete filling of the micro-cavities of the PDMS master mold by collagen gel. As the compressive pressure increased to 50 and 115 kPa, the top of the micro-ridge patterns widened, and the edges became less rounded (Figure 2(ai)). In the dried state, the IPC-CE constructs clearly exhibited micro-ridge patterns on their bottom region, while the collagen structure of the top region collapsed over the bottom surface during the drying process. The micro-ridge patterns on the bottom regions were well transcribed from the PDMS master mold and showed clear borderlines under a UV dose of 3.0 J/cm^2^, regardless of the compressive pressure applied (except at 2 kPa; Figure 2(aii)).

The formation of micro-ridge patterns on the IPC-CE construct was also found to be significantly affected by the UV dose, while a constant compressive pressure (115 kPa) was applied during the IPC-CE process. The narrow tops and rounded edges of the micro-ridge patterns were observed on the construct subjected to the lowest UV dose (0.90 J/cm^2^), while the top area gradually widened as the UV dose increased to 3.00 J/cm^2^ (Figure 2(bi)). The micro-patterns on the bottom region of the dried construct showed a high micro-pattern fidelity under a compressive pressure of 115 kPa, regardless of the UV dose (Figure 2(bii)). By measuring the widths at each top and bottom of the micro-ridge patterns, it was found that the top width of the micro-ridge patterns gradually increased from 20.3 ± 6.2 μm to 71.9 ± 12.2 μm and from 18.7 ± 7.3 μm to 71.9 ± 12.2 μm as the compressive pressure and the UV dose increased from 13 to 115 kPa and from 0.90 to 3.00 J/cm^2^, respectively, while maintaining the same bottom width (≈98–99 μm) (Figure 2c).

### 3.3. Nanofibrillar Architecture of the IPC-CE Construct

**Figure 3:**
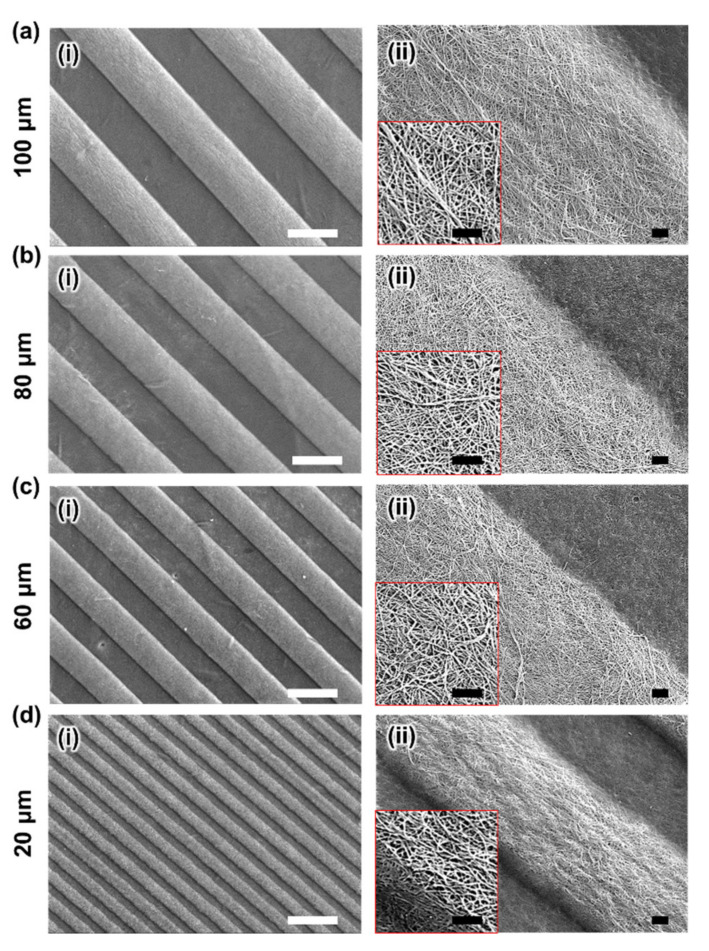
Microstructures of the IPC-CE constructs. SEM images of micro-ridge patterns with different widths of (**a**) 100, (**b**) 80, (**c**) 60, and (**d**) 20 μm at (**i**) low and (**ii**) high magnifications. White and black scale bars are 100 and 2 μm, respectively.

The SEM analysis clearly showed that the micro-patterns of the IPC-CE construct included both micro-ridge patterns and collagen nanofibrillar architectures, resulting in the formation of hierarchical micro/nano topographical structures, taking advantage of using the collagen gel. The nanofibrillar collagen micro-ridge patterns (widths of 100, 80, 60, and 20 μm) without any noticeable defect were favorably formed during the IPC-CE process; moreover, various types of nanofibrillar collagen micro-patterns (e.g., micro-pillar and fingerprint patterns) could be generated depending on the PDMS master molds used (Figure 3 and Figure S2). The highly magnified SEM images showed randomly oriented and homogeneous dense collagen nanofibrillar architectures (fibril diameter = ≈100 nm) located inside the micro-patterns. 

### 3.4. Structural Robustness and Topographical Effect of the IPC-CE Construct in an in Vitro Cell Culture

**Figure 4:**
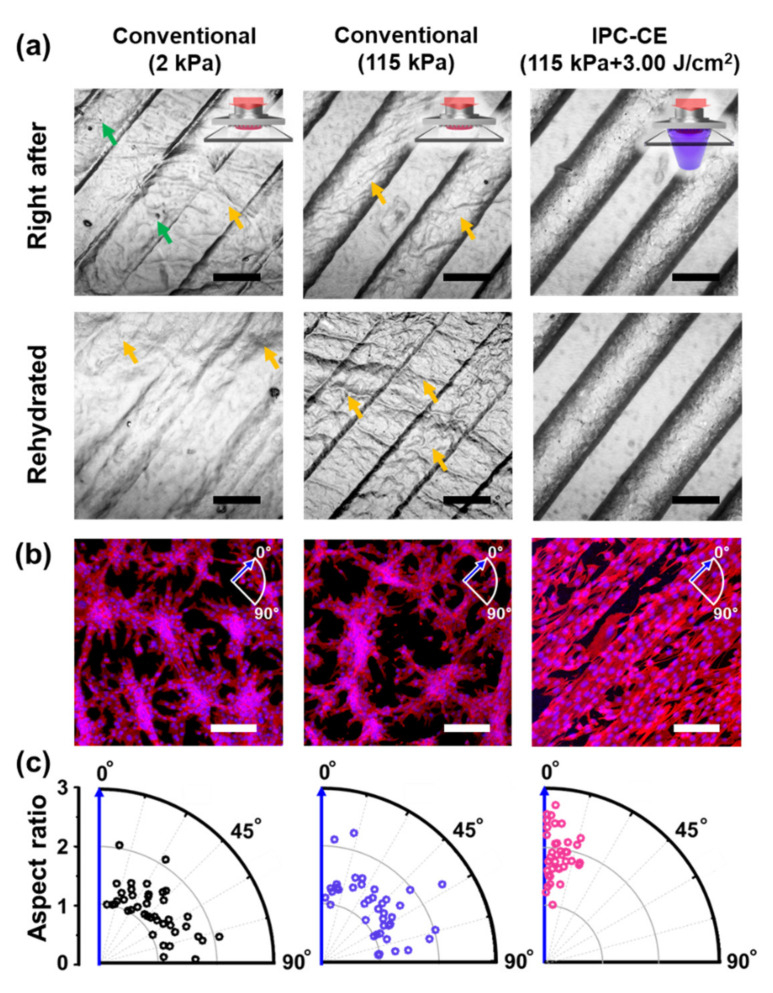
Robustness and topographical effect of the IPC-CE construct. (**a**) Microscopic observation of conventional (2 and 115 kPa without UV dose) and IPC-CE construct, right after fabrication and after immersion in PBS solution. Green and yellow arrows indicate defects by partial replication and wrinkled structure, respectively. (**b**) NIH 3T3 cells cultured on each construct on day 2 (DAPI, blue; phalloidin, red). (**c**) Aspect ratio and orientation of NIH 3T3 cells. Blue arrow indicates direction of micro-ridge patterns. All scale bars are 100 μm.

The structural robustness of the micro-patterns on the IPC-CE construct subjected to rehydration was assessed by investigating the temporal changes of the micro-ridge patterns in a PBS solution, as well as the corresponding topographical effect on cells, and comparing it with the robustness of micro-patterns on a conventional construct. Figure 4a shows the structural change of the micro-patterns on both the IPC-CE and the conventional constructs in the PBS solution. Similarly to what was observed in Figure 2a, right after the collagen compression-based embossing under a compressive pressure of 2 kPa, the conventional construct showed partially replicated micro-ridge patterns; additionally, frequently discontinuous and undesirably wrinkled structures induced by the manual handling were observed across the entire surface of the construct. Notably, the micro-patterns observed on the 2 kPa-conventional construct disappeared within 10 min of rehydration in the PBS solution. Although a high compressive pressure (115 kPa) was found to improve the transcription quality of the micro-patterns (to levels similar to those obtained through the IPC-CE process), a 10-min rehydration of the sample induced the removal of the micro-ridge patterns, while leaving footprints at their edges and random wrinkling. Meanwhile, the IPC-CE construct remarkably maintained its robust micro-ridge patterns without showing wrinkling in the PBS solution; moreover, no evidence of structural disruption was found even after several days of rehydration in the PBS solution. 

The alignment of the NIH 3T3 cells in response to the topographical effect of the micro-ridge patterns further demonstrated the structural robustness of the micro-patterns generated by the IPC-CE process (Figure 4b). Although the cells favorably attached and proliferated on all the experimental groups (owing to the cell-friendly collagen surface), the F-actin (phalloidin, red) and nucleus (DAPI, blue) immunofluorescence staining images showed remarkable differences in terms of cell orientation and aspect ratio between the conventional and IPC-CE constructs. After 2 days of cell culture, the cells attached on two different conventional constructs (fabricated at 2 and 115 kPa) showed a well-spread morphology and random orientations, without a noticeable topographical effect. This can be explained as an effect of rehydration. On the contrary, the micro-ridge patterns of the IPC-CE construct were found to effectively induce both the elongation and uniaxial alignment of cells along the patterns due to topographical effect, even in the rehydration state. The quantitative analysis of cell nuclei (Figure 4c) further confirmed that those of cells on the IPC-CE construct had a relatively high aspect ratio (≈1.9) and a small deviation angle (<20°) from the micro-ridge pattern, while the cells on the conventional constructs showed a relatively low aspect ratio (≈1.4), an arbitrary orientation, and a large deviation angle.

## 4. Discussion

The self-assembly process based on non-covalent molecular interactions, such as hydrophobic forces, electrostatic interactions, and hydrogen bonding, is one of the powerful methods to produce in vivo-like nanofibrils as a bottom–up approach [26,27,28,29]. The self-assembled nanofibrillar collagen constructs have been previously found to provide the best environment for cell culture; however, one of the major concerns with respect to the micro-patterning of the nanofibrillar collagen gel is the structural robustness of the resultant micro-patterns in aqueous media. According to the previous study, the microscale patterning process required an adequate elastic modulus of base material on the level of 0.1–1 MPa to acquire the desired transcription quality [30]. While collagen compression-based embossing has provided a facile and efficient way to produce micro-patterned collagen constructs along with increasing the elastic modulus of the construct to the required level of 0.1–1 MPa [18,31], the correspondent collagen micro-patterns can be seriously deteriorated by external factors (e.g., rehydration and manual handling). Notably, damaged collagen micro-patterns lose their topographical effect on cells, hampering the practical utilization of collagen constructs as cell culture substrates for in vitro model or scaffold tissue regeneration. One of our recent studies revealed that compressed collagen constructs can suffer from severe expansion (≈3 times) due to rehydration, since this latter process induces sparsely distributed collagen nanofibrillar structures in the construct [32]. This means that the collagen micro-patterns can be easily disrupted along with the reorganization of the collagen nanofibrils induced by rehydration. Here, we demonstrated for the first time that collagen micro-patterns generated through the collagen compression-based embossing process tend to rapidly disappear in an aqueous solution within 10 min, leaving behind their traces. An in situ photochemical crosslinking (IPC), conducted during the IPC-CE process, allowed the acquisition of well-replicated and highly robust collagen micro-patterns, which maintained their collagen nanofibrillar architectures in the aqueous medium for a long period of time. It has been recently demonstrated that the IPC, when conducted during the collagen compression process, can efficiently lead to the formation of chemical bonds within densely packed collagen nanofibrillar structures under compressive pressure, significantly increasing the mechanical properties of the collagen construct and avoiding rehydration [32]. Similarly, when the IPC was applied during the proposed collagen compression-based embossing, it enabled the formation of covalently bonded collagen nanofibrillar architecture within the collagen micro-patterns, which were robust enough to resist under rehydration and manual handling. 

In addition, the IPC-CE process accomplished versatility in the micro-patterning of the nanofibrillar collagen gel by enabling the utilization of the PDMS master mold prepared with the simple replication process. Conventional collagen compression-based embossing generally relied on a specific phenomenon, where the collagen fibrils are more highly compacted at the interface between the collagen gel and the absorbent material used for dehydration, which limited the geometry of fabricated collagen micro-patterns [18,21]. Thus, to make a specific collagen micro-pattern, it is necessary to prepare a specially designed micro-patterned template with proper water absorbability/permeability, which would enable the dehydration of the collagen gel at specific positions. The results of our parametric analyses found that the IPC-CE process can lead to the production of collagen micro-patterns with favorable transcription qualities under proper compressive pressures and UV doses, despite the use of a PDMS master mold that was not ideal for dehydration. The results indicated that a higher compressive pressure can promote the filling of the collagen fibrils into the micro-patterned PDMS master mold and a proper UV dose concurrently applied during the compression finally determined the robustness of the micro-patterns. All these data demonstrated the potential of the IPC-CE process in generating collagen micro-patterns of various sizes and shapes (minimum feature size <10 μm) in a facile way.

The investigation of the in vitro NIH 3T3 cell culture described in the paper confirmed the practical applicability of the proposed IPC-CE construct for biomedical applications, as well as its structural robustness and the topographical effect of the micro-ridge patterns. Many previous studies have shown that the NIH 3T3 cells tend to be elongated and oriented along anisotropic topographies, as a result of contact guidance by nanoscale patterns or geometrical confinement by microscale patterns (>10 μm) [6,33,34,35]. Our results showed that the micro-ridge patterns on the IPC-CE construct favorably promoted both the elongation and orientation of cells along the micro-ridges by geometrical confinement, which stably maintained their structure during the cell culture period, whereas the conventional constructs lost their topographical effect following rehydration. Although in the present study, we considered only a simple in vitro cell culture, the proposed IPC-CE construct may have various biomedical applications, such as the development of an in vitro model for mimicking the hierarchical topography of native tissues and a scaffold/wound dressing for promoting in vivo tissue regeneration [36,37]. Moreover, given that the attachment, alignment, and elongation of cells resulted from their sensing and response to the surrounding cell environment [38,39], a more profound understanding of in vivo cell-surface topography interactions may be accomplished from in-depth investigations of cell behaviors on micro-patterned constructs with in vivo-like collagen nanofibrillar architectures.

## 5. Conclusions

This study suggests a novel integrative fabrication technique, named IPC-CE process, for the robust micro-patterning of nanofibrillar collagen hydrogel. Through this process, it was possible to produce robust collagen micro-patterns by applying photochemical crosslinking in situ during collagen compression-based embossing, eventually obtaining a product with high resistivity against rehydration and manual handling. The IPC-CE process allowed the fabrication of a versatile IPC-CE construct with topographical micro-patterns of various sizes and shapes. The practical applicability of the IPC-CE construct was finally demonstrated by applying it to an in vitro NIH 3T3 cell culture: the collagen micro-patterns maintained their topographical effect during the cell culture. Overall, our results validate a new method for the fabrication of functional collagen constructs with topographical micro-patterns and support their application in the fields of tissue engineering and regenerative medicine.

## Supplementary Materials

The following are available online at https://www.mdpi.com/2079-4991/10/12/2574/s1, Figure S1: Conventional collagen compression-based embossing and IPC-CE process. Figure S2: Various sizes and shapes of micro-patterns.
