# Supplementary material for: Robust Topographical Micro-Patterning of Nanofibrillar Collagen Gel by In Situ Photochemical Crosslinking-Assisted Collagen Embossing
Source: Nanomaterials (Basel). 2020 Dec 21;10(12):2574. doi: 10.3390/nano10122574 (PMC7767481; doi:10.3390/nano10122574)
Supplement: Supplementary file 1 [file nanomaterials-10-02574-s001.pdf]

Supplementary information

Robust topographical micro-patterning of nanofibrillar collagen gel by *in situ* photochemical crosslinking-assisted collagen embossing

Hyeonjun Hong and Dong Sung Kim

(1) Conventional collagen compression-based embossing and IPC-CE process

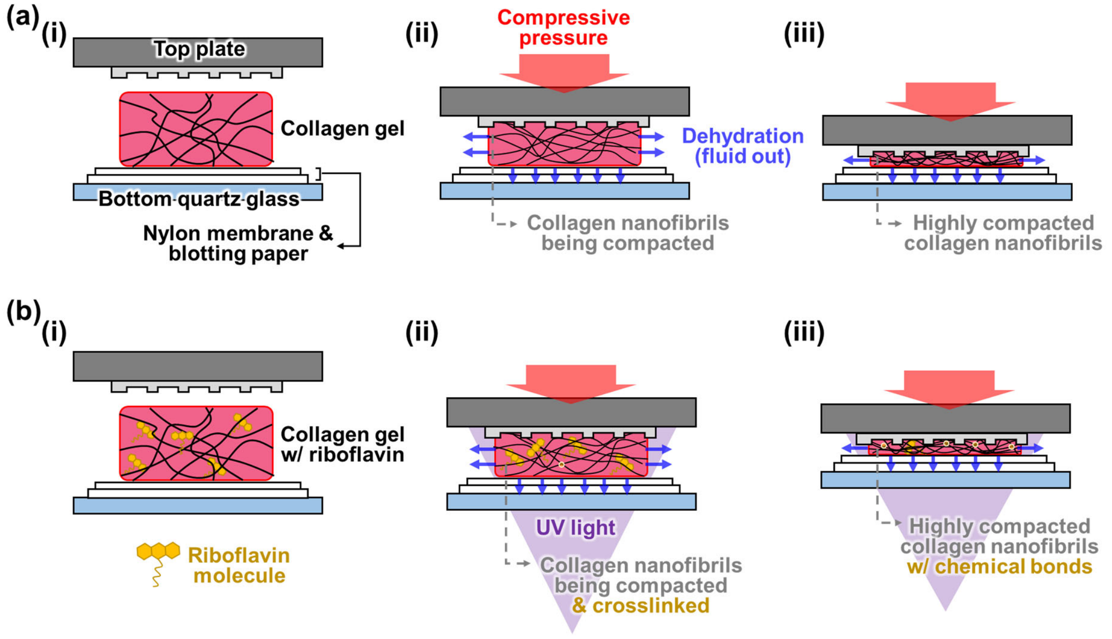

Figure S1. Schematic illustrations of (a) Conventional compression-based embossing and (b) IPC-CE process. Collagen gel (i) at initial, (ii) intermediate, and (iii) final stage of each process.

(2) Various sizes and shapes of micro-patterns

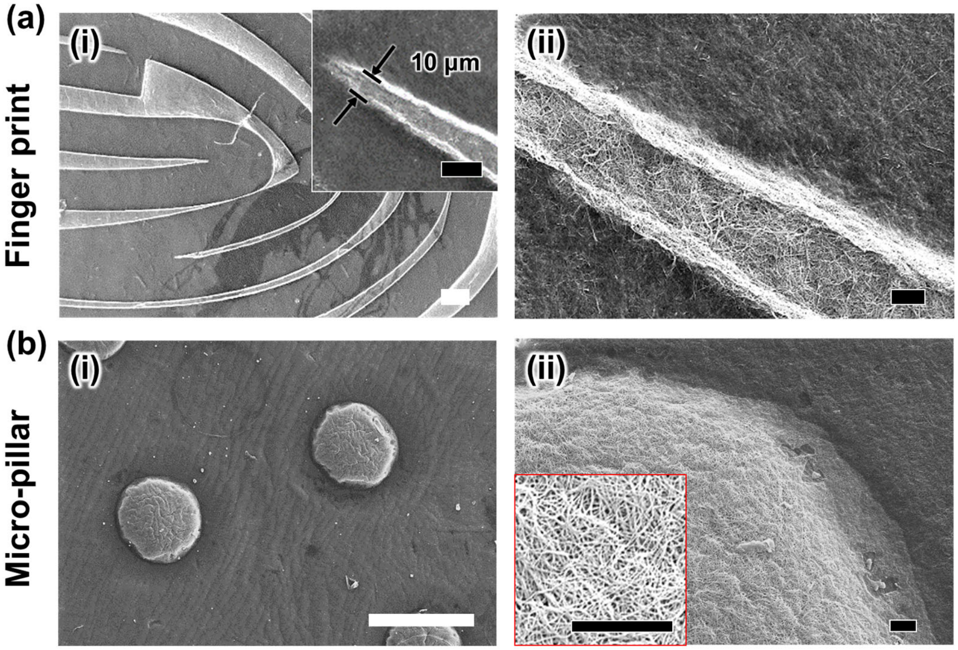

Figure S2. Various sizes and shapes of micro-patterns of IPC-CE construct. SEM images of IPC-CE construct with (a) fingerprint (~ 8–700 μm in width) and (b) micro-pillar patterns (~ 150 μm in diameter) at (i) low and (ii) high magnifications. White and black scale bars are 200 and 5 μm, respectively.
